# *N*-(4-Methyl­benzo­yl)-2-nitro­benzene­sulfonamide

**DOI:** 10.1107/S1600536812003522

**Published:** 2012-02-04

**Authors:** P. A. Suchetan, Sabine Foro, B. Thimme Gowda

**Affiliations:** a Department of Chemistry, Mangalore University, Mangalagangotri 574 199, Mangalore, India; b Institute of Materials Science, Darmstadt University of Technology, Petersenstrasse 23, D-64287 Darmstadt, Germany

## Abstract

The asymmetric unit of the title compound, C_14_H_12_N_2_O_5_S, contains two independent mol­ecules. The dihedral angles between the aromatic rings are 82.03 (9) and 79.47 (8)° in the two independent mol­ecules. In the crystal, the two mol­ecules in the asymmetric unit are linked into dimers *via* pairs of N—H⋯O(S) hydrogen bonds to generate *C*(4) chains.

## Related literature

For studies, including ours, on the effects of substituents on the structures and other aspects of *N*-(ar­yl)amides, see: Bowes *et al.* (2003 ▶); Gowda *et al.* (1999 ▶, 2003 ▶). For *N*-(ar­yl)methane­sulfonamides, see: Gowda *et al.* (2007 ▶). For *N*-(ar­yl)aryl­sulfonamides, see: Shetty & Gowda (2005 ▶). For *N*-(sub­stituted benzo­yl)aryl­sulfonamides, see: Suchetan *et al.* (2012 ▶). For *N*-chloro­aryl­amides, see: Jyothi & Gowda (2004 ▶). For *N*-bromo­aryl­sulfonamides, see: Usha & Gowda (2006 ▶).
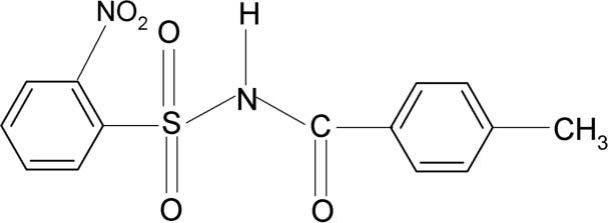


## Experimental

### 

#### Crystal data

C_14_H_12_N_2_O_5_S
*M* *_r_* = 320.32
Triclinic, 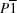

*a* = 10.860 (1) Å
*b* = 11.716 (2) Å
*c* = 12.841 (2) Å
α = 114.51 (2)°
β = 102.99 (2)°
γ = 91.16 (1)°
*V* = 1436.6 (4) Å^3^
*Z* = 4
Mo *K*α radiation
μ = 0.25 mm^−1^
*T* = 293 K
0.44 × 0.44 × 0.24 mm

#### Data collection

Oxford Diffraction Xcalibur diffractometer with a Sapphire CCD detector
Absorption correction: multi-scan (*CrysAlis RED*; Oxford Diffraction, 2009 ▶) *T*_min_ = 0.898, *T*_max_ = 0.942
8993 measured reflections
5772 independent reflections
4069 reflections with *I* > 2σ(*I*)
*R*_int_ = 0.016

#### Refinement

*R*[*F* ^2^ > 2σ(*F* ^2^)] = 0.049
*wR*(*F* ^2^) = 0.116
*S* = 1.06
5772 reflections
405 parameters
2 restraints
H atoms treated by a mixture of independent and constrained refinement
Δρ_max_ = 0.29 e Å^−3^
Δρ_min_ = −0.31 e Å^−3^

### 

Data collection: *CrysAlis CCD* (Oxford Diffraction, 2009 ▶); cell refinement: *CrysAlis RED* (Oxford Diffraction, 2009 ▶); data reduction: *CrysAlis RED*; program(s) used to solve structure: *SHELXS97* (Sheldrick, 2008 ▶); program(s) used to refine structure: *SHELXL97* (Sheldrick, 2008 ▶); molecular graphics: *PLATON* (Spek, 2009 ▶); software used to prepare material for publication: *SHELXL97*.

## Supplementary Material

Crystal structure: contains datablock(s) I, global. DOI: 10.1107/S1600536812003522/bt5801sup1.cif

Structure factors: contains datablock(s) I. DOI: 10.1107/S1600536812003522/bt5801Isup2.hkl

Supplementary material file. DOI: 10.1107/S1600536812003522/bt5801Isup3.cml

Additional supplementary materials:  crystallographic information; 3D view; checkCIF report

## Figures and Tables

**Table 1:** Hydrogen-bond geometry (Å, °)

*D*—H⋯*A*	*D*—H	H⋯*A*	*D*⋯*A*	*D*—H⋯*A*
N3—H3N⋯O2	0.85 (1)	2.30 (1)	3.141 (3)	168 (3)

**Fig. 1.**
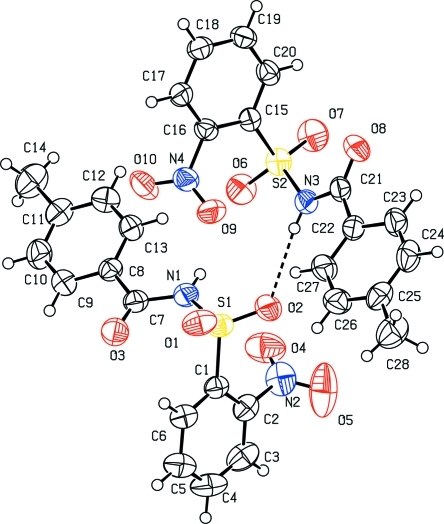
Molecular structure of the title compound, showing the atom-labelling scheme. Displacement ellipsoids are drawn at the 50% probability level.

**Fig. 2.**
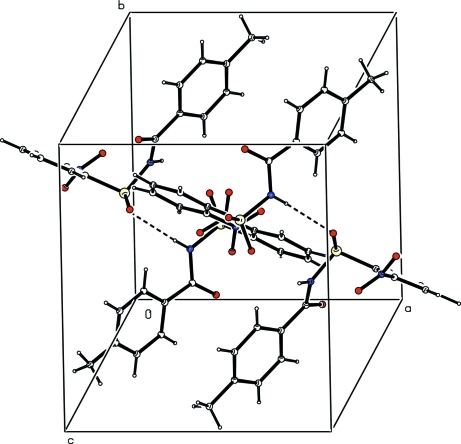
Molecular packing in the title compound. Hydrogen bonds are shown as dashed lines.

## supplementary crystallographic information

### Comment

Diaryl acylsulfonamides are known as potent antitumor agents. As part of our studies on the substituent effects on the structures and other aspects of *N*-(aryl)-amides (Bowes *et al.*, 2003; Gowda *et al.*, 1999, 2003), *N*-(aryl)-methanesulfonamides (Gowda *et al.*, 2007), *N*-(aryl)-arylsulfonamides (Shetty & Gowda, 2005); *N*-(substitutedbenzoyl)-arylsulfonamides (Suchetan *et al.*, 2012); *N*-chloroarylsulfonamides (Jyothi & Gowda, 2004) and *N*-bromoarylsulfonamides (Usha & Gowda, 2006), in the present work, the crystal structure of *N*-(4-methylbenzoyl)-2-nitrobenzenesulfonamide (I) has been determined (Fig.1).

The asymmetric unit of the structure contains two independent molecules. In one of the molecules, the N—C bond in the C—SO_2_—NH—C segment has *gauche* torsion with respect to the S═O bonds. The conformation between the N—H and C=O bonds in the C—SO_2_—NH—C(O) segments are *anti*. Further, the conformations between the N—H bonds and the *ortho*-nitro groups in the sulfonyl benzene rings are *syn*, similar to that observed in *N*-(3-methylbenzoyl)- 2-nitrobenzenesulfonamide (II)(Suchetan *et al.*, 2012).

The molecules are twisted at the S—N bonds with the torsional angles of 64.42 (25)° and -55.37 (245)°, compared to the value of 64.32 (20)° in (II).

The dihedral angles between the sulfonyl benzene rings and the —SO_2_—NH—C—O segments are 76.73 (8)° and 79.47 (8)°, compared to the value of 75.7 (1)° in (II). Furthermore, the dihedral angles between the sulfonyl and the benzoyl benzene rings are 82.03 (9)° and 79.47 (8)°, compared to the value of 89.5 (1)° in (II).

In the crystal, the intermolecular N–H···O (S) hydrogen bonds (Table 1) link the molecules into chains. Part of the crystal structure is shown in Fig. 2.

### Experimental

The title compound was prepared by refluxing a mixture of *p*-methylbenzoic acid (0.02 mole), 2-nitrobenzenesulfonamide (0.02 mole) and excess phosphorous oxychloride for 3 h on a water bath. The resultant mixture was cooled and poured into crushed ice. The solid, *N*-(4-methylbenzoyl)-2-nitrobenzenesulfonamide, obtained was filtered, washed thoroughly with water and then dissolved in sodium bicarbonate solution. The compound was later reprecipitated by acidifying the filtered solution with dilute HCl. It was filtered, dried and recrystallized.

Prism like colourless single crystals of the title compound used in X-ray diffraction studies were obtained by slow evaporation of its toluene solution at room temperature.

### Refinement

The H atom of the NH group was located in a difference map and later restrained to N—H = 0.86 (2) Å. The other H atoms were positioned with idealized geometry using a riding model with the aromatic C—H = 0.93 Å and the methyl C—H = 0.93 Å. All H atoms were refined with isotropic displacement parameters set at 1.2 *U*_eq_(C-aromatic, N) and 1.5 *U*_eq_(C-methyl).

### Crystal data

**Table:**

C_14_H_12_N_2_O_5_S	*Z* = 4
*M**_r_* = 320.32	*F*(000) = 664
Triclinic, *P*1	*D*_x_ = 1.481 Mg m^−^^3^
Hall symbol: -P 1	Mo *K*α radiation, λ = 0.71073 Å
*a* = 10.860 (1) Å	Cell parameters from 3497 reflections
*b* = 11.716 (2) Å	θ = 2.6–27.7°
*c* = 12.841 (2) Å	µ = 0.25 mm^−^^1^
α = 114.51 (2)°	*T* = 293 K
β = 102.99 (2)°	Prism, colourless
γ = 91.16 (1)°	0.44 × 0.44 × 0.24 mm
*V* = 1436.6 (4) Å^3^	

### Data collection

**Table:**

Oxford Diffraction Xcalibur diffractometer with a Sapphire CCD detector	5772 independent reflections
Radiation source: fine-focus sealed tube	4069 reflections with *I* > 2σ(*I*)
Graphite monochromator	*R*_int_ = 0.016
Rotation method data acquisition using ω scans	θ_max_ = 26.4°, θ_min_ = 2.6°
Absorption correction: multi-scan (*CrysAlis RED*; Oxford Diffraction, 2009)	*h* = −13→13
*T*_min_ = 0.898, *T*_max_ = 0.942	*k* = −12→14
8993 measured reflections	*l* = −14→16

### Refinement

**Table:**

Refinement on *F*^2^	Primary atom site location: structure-invariant direct methods
Least-squares matrix: full	Secondary atom site location: difference Fourier map
*R*[*F*^2^ > 2σ(*F*^2^)] = 0.049	Hydrogen site location: inferred from neighbouring sites
*wR*(*F*^2^) = 0.116	H atoms treated by a mixture of independent and constrained refinement
*S* = 1.06	*w* = 1/[σ^2^(*F*_o_^2^) + (0.0321*P*)^2^ + 1.1761*P*] where *P* = (*F*_o_^2^ + 2*F*_c_^2^)/3
5772 reflections	(Δ/σ)_max_ = 0.018
405 parameters	Δρ_max_ = 0.29 e Å^−^^3^
2 restraints	Δρ_min_ = −0.31 e Å^−^^3^

### Special details

**Table:**

Experimental. CrysAlis RED (Oxford Diffraction, 2009) Empirical absorption correction using spherical harmonics, implemented in SCALE3 ABSPACK scaling algorithm.
Geometry. All e.s.d.'s (except the e.s.d. in the dihedral angle between two l.s. planes) are estimated using the full covariance matrix. The cell e.s.d.'s are taken into account individually in the estimation of e.s.d.'s in distances, angles and torsion angles; correlations between e.s.d.'s in cell parameters are only used when they are defined by crystal symmetry. An approximate (isotropic) treatment of cell e.s.d.'s is used for estimating e.s.d.'s involving l.s. planes.
Refinement. Refinement of *F*^2^ against ALL reflections. The weighted *R*-factor *wR* and goodness of fit *S* are based on *F*^2^, conventional *R*-factors *R* are based on *F*, with *F* set to zero for negative *F*^2^. The threshold expression of *F*^2^ > σ(*F*^2^) is used only for calculating *R*-factors(gt) *etc*. and is not relevant to the choice of reflections for refinement. *R*-factors based on *F*^2^ are statistically about twice as large as those based on *F*, and *R*- factors based on ALL data will be even larger.

### Fractional atomic coordinates and isotropic or equivalent isotropic displacement parameters (Å^2^)

**Table:**

	*x*	*y*	*z*	*U*_iso_*/*U*_eq_	
C1	−0.0965 (2)	0.5718 (2)	0.3025 (2)	0.0424 (6)	
C2	−0.1921 (3)	0.5564 (2)	0.2042 (3)	0.0485 (6)	
C3	−0.3051 (3)	0.6057 (3)	0.2163 (3)	0.0642 (9)	
H3	−0.3696	0.5933	0.1498	0.077*	
C4	−0.3212 (3)	0.6736 (3)	0.3285 (4)	0.0713 (10)	
H4	−0.3959	0.7095	0.3375	0.086*	
C5	−0.2284 (3)	0.6885 (3)	0.4263 (3)	0.0661 (9)	
H5	−0.2405	0.7333	0.5015	0.079*	
C6	−0.1166 (3)	0.6369 (3)	0.4134 (3)	0.0505 (7)	
H6	−0.0541	0.6462	0.4802	0.061*	
C7	0.1874 (2)	0.7296 (3)	0.3702 (2)	0.0467 (6)	
C8	0.2763 (2)	0.8070 (2)	0.3448 (2)	0.0428 (6)	
C9	0.2955 (3)	0.9366 (3)	0.4126 (3)	0.0587 (8)	
H9	0.2529	0.9730	0.4714	0.070*	
C10	0.3781 (3)	1.0120 (3)	0.3926 (3)	0.0659 (9)	
H10	0.3898	1.0990	0.4384	0.079*	
C11	0.4436 (3)	0.9617 (3)	0.3068 (3)	0.0545 (7)	
C12	0.4233 (3)	0.8330 (3)	0.2396 (3)	0.0525 (7)	
H12	0.4656	0.7971	0.1805	0.063*	
C13	0.3409 (3)	0.7557 (3)	0.2582 (2)	0.0473 (6)	
H13	0.3292	0.6688	0.2121	0.057*	
C14	0.5351 (3)	1.0447 (3)	0.2876 (3)	0.0790 (10)	
H14A	0.6115	1.0720	0.3509	0.095*	
H14B	0.4966	1.1172	0.2861	0.095*	
H14C	0.5556	0.9979	0.2136	0.095*	
N1	0.1440 (2)	0.6072 (2)	0.2821 (2)	0.0488 (5)	
H1N	0.157 (3)	0.583 (3)	0.2132 (13)	0.059*	
N2	−0.1779 (3)	0.4904 (3)	0.0836 (3)	0.0704 (8)	
O1	0.09629 (18)	0.4991 (2)	0.40379 (19)	0.0633 (6)	
O2	0.02871 (19)	0.39485 (17)	0.18596 (19)	0.0601 (5)	
O3	0.1506 (2)	0.7687 (2)	0.45992 (18)	0.0659 (6)	
O4	−0.0976 (3)	0.5353 (3)	0.0563 (2)	0.0944 (8)	
O5	−0.2522 (4)	0.3971 (3)	0.0170 (2)	0.1311 (13)	
S1	0.04803 (6)	0.50626 (6)	0.29466 (7)	0.04812 (18)	
C15	0.4933 (2)	0.2942 (2)	0.1683 (2)	0.0380 (5)	
C16	0.5306 (2)	0.3732 (2)	0.2893 (2)	0.0414 (6)	
C17	0.6504 (2)	0.3816 (3)	0.3571 (2)	0.0468 (6)	
H17	0.6740	0.4362	0.4374	0.056*	
C18	0.7355 (3)	0.3071 (3)	0.3037 (3)	0.0503 (7)	
H18	0.8171	0.3115	0.3485	0.060*	
C19	0.7009 (3)	0.2267 (3)	0.1852 (3)	0.0507 (7)	
H19	0.7585	0.1757	0.1506	0.061*	
C20	0.5805 (3)	0.2213 (2)	0.1172 (2)	0.0477 (6)	
H20	0.5583	0.1683	0.0365	0.057*	
C21	0.2377 (3)	0.0971 (2)	0.0854 (2)	0.0479 (6)	
C22	0.1465 (2)	0.0519 (2)	0.1349 (2)	0.0457 (6)	
C23	0.1197 (3)	−0.0777 (3)	0.0968 (3)	0.0570 (7)	
H23	0.1556	−0.1334	0.0392	0.068*	
C24	0.0401 (3)	−0.1238 (3)	0.1443 (3)	0.0623 (8)	
H24	0.0222	−0.2108	0.1168	0.075*	
C25	−0.0139 (3)	−0.0445 (3)	0.2314 (3)	0.0572 (7)	
C26	0.0137 (3)	0.0836 (3)	0.2686 (3)	0.0613 (8)	
H26	−0.0217	0.1391	0.3268	0.074*	
C27	0.0925 (3)	0.1320 (3)	0.2217 (3)	0.0560 (7)	
H27	0.1093	0.2191	0.2487	0.067*	
C28	−0.0969 (3)	−0.0950 (3)	0.2860 (4)	0.0813 (10)	
H28A	−0.0514	−0.1478	0.3175	0.098*	
H28B	−0.1728	−0.1437	0.2267	0.098*	
H28C	−0.1197	−0.0257	0.3486	0.098*	
N3	0.2353 (2)	0.2211 (2)	0.0981 (2)	0.0467 (5)	
H3N	0.185 (2)	0.269 (2)	0.133 (2)	0.056*	
N4	0.4405 (2)	0.4484 (2)	0.3514 (2)	0.0524 (6)	
O6	0.3190 (2)	0.42572 (18)	0.11978 (19)	0.0637 (6)	
O7	0.3564 (2)	0.2349 (2)	−0.04299 (17)	0.0708 (6)	
O8	0.3129 (2)	0.03374 (18)	0.0378 (2)	0.0677 (6)	
O9	0.3422 (2)	0.3918 (2)	0.34577 (19)	0.0780 (7)	
O10	0.4714 (2)	0.5617 (2)	0.4097 (2)	0.0713 (6)	
S2	0.34704 (7)	0.29805 (7)	0.07566 (6)	0.04910 (19)	

### Atomic displacement parameters (Å^2^)

**Table:**

	*U*^11^	*U*^22^	*U*^33^	*U*^12^	*U*^13^	*U*^23^
C1	0.0391 (14)	0.0388 (13)	0.0565 (16)	0.0053 (11)	0.0140 (12)	0.0266 (12)
C2	0.0499 (16)	0.0463 (15)	0.0566 (17)	0.0015 (12)	0.0095 (13)	0.0316 (14)
C3	0.0475 (17)	0.076 (2)	0.083 (2)	0.0057 (15)	0.0037 (16)	0.055 (2)
C4	0.0478 (18)	0.091 (2)	0.103 (3)	0.0269 (17)	0.0312 (19)	0.062 (2)
C5	0.060 (2)	0.075 (2)	0.078 (2)	0.0204 (17)	0.0329 (18)	0.0380 (19)
C6	0.0441 (15)	0.0573 (17)	0.0577 (18)	0.0087 (13)	0.0150 (13)	0.0309 (14)
C7	0.0390 (14)	0.0520 (16)	0.0475 (16)	0.0079 (12)	0.0113 (12)	0.0198 (13)
C8	0.0377 (13)	0.0456 (15)	0.0452 (15)	0.0063 (11)	0.0072 (11)	0.0214 (12)
C9	0.0572 (18)	0.0503 (17)	0.0599 (19)	0.0060 (14)	0.0195 (15)	0.0132 (14)
C10	0.068 (2)	0.0407 (16)	0.081 (2)	0.0025 (15)	0.0139 (18)	0.0211 (16)
C11	0.0503 (16)	0.0547 (17)	0.0661 (19)	0.0029 (13)	0.0075 (14)	0.0373 (15)
C12	0.0562 (17)	0.0580 (17)	0.0555 (17)	0.0113 (14)	0.0205 (14)	0.0329 (15)
C13	0.0536 (16)	0.0420 (14)	0.0487 (16)	0.0073 (12)	0.0136 (13)	0.0216 (12)
C14	0.079 (2)	0.073 (2)	0.100 (3)	−0.0053 (19)	0.018 (2)	0.054 (2)
N1	0.0477 (13)	0.0477 (13)	0.0526 (14)	0.0023 (10)	0.0190 (11)	0.0202 (12)
N2	0.084 (2)	0.0717 (19)	0.0577 (18)	0.0124 (16)	0.0071 (16)	0.0360 (16)
O1	0.0498 (12)	0.0827 (15)	0.0815 (15)	0.0207 (11)	0.0173 (11)	0.0576 (13)
O2	0.0604 (13)	0.0395 (10)	0.0803 (15)	0.0126 (9)	0.0240 (11)	0.0224 (10)
O3	0.0666 (14)	0.0700 (14)	0.0563 (13)	−0.0036 (11)	0.0273 (11)	0.0171 (11)
O4	0.0899 (19)	0.132 (2)	0.0839 (19)	0.0306 (18)	0.0437 (16)	0.0570 (18)
O5	0.191 (4)	0.098 (2)	0.0616 (18)	−0.042 (2)	−0.013 (2)	0.0194 (16)
S1	0.0431 (4)	0.0460 (4)	0.0649 (5)	0.0112 (3)	0.0174 (3)	0.0311 (3)
C15	0.0444 (14)	0.0359 (13)	0.0373 (13)	0.0020 (11)	0.0130 (11)	0.0180 (11)
C16	0.0447 (14)	0.0406 (14)	0.0440 (15)	0.0077 (11)	0.0192 (12)	0.0189 (12)
C17	0.0442 (15)	0.0498 (15)	0.0416 (15)	0.0018 (12)	0.0118 (12)	0.0150 (12)
C18	0.0403 (15)	0.0529 (16)	0.0591 (18)	0.0062 (12)	0.0140 (13)	0.0249 (14)
C19	0.0490 (16)	0.0483 (15)	0.0584 (18)	0.0124 (13)	0.0251 (14)	0.0205 (14)
C20	0.0583 (17)	0.0423 (14)	0.0434 (15)	0.0048 (12)	0.0208 (13)	0.0153 (12)
C21	0.0466 (15)	0.0353 (14)	0.0478 (16)	0.0053 (12)	0.0049 (12)	0.0081 (12)
C22	0.0418 (14)	0.0371 (13)	0.0495 (15)	0.0060 (11)	0.0019 (12)	0.0152 (12)
C23	0.0618 (18)	0.0384 (15)	0.0591 (18)	0.0053 (13)	0.0095 (15)	0.0128 (13)
C24	0.068 (2)	0.0386 (15)	0.071 (2)	−0.0025 (14)	0.0025 (17)	0.0222 (15)
C25	0.0461 (16)	0.0550 (17)	0.069 (2)	0.0004 (13)	0.0009 (14)	0.0326 (16)
C26	0.0589 (18)	0.0525 (17)	0.076 (2)	0.0148 (14)	0.0243 (16)	0.0271 (16)
C27	0.0614 (18)	0.0370 (14)	0.071 (2)	0.0116 (13)	0.0217 (16)	0.0219 (14)
C28	0.072 (2)	0.077 (2)	0.099 (3)	−0.0049 (19)	0.017 (2)	0.046 (2)
N3	0.0446 (13)	0.0407 (12)	0.0497 (14)	0.0066 (10)	0.0092 (10)	0.0160 (11)
N4	0.0483 (14)	0.0625 (16)	0.0407 (13)	0.0112 (12)	0.0136 (11)	0.0155 (12)
O6	0.0703 (14)	0.0520 (12)	0.0798 (15)	0.0140 (10)	0.0135 (11)	0.0415 (11)
O7	0.0799 (15)	0.0911 (16)	0.0404 (11)	0.0088 (13)	0.0109 (10)	0.0296 (11)
O8	0.0688 (14)	0.0444 (11)	0.0808 (15)	0.0134 (10)	0.0312 (12)	0.0119 (10)
O9	0.0462 (12)	0.0970 (18)	0.0665 (15)	−0.0018 (12)	0.0232 (11)	0.0081 (13)
O10	0.0874 (16)	0.0527 (13)	0.0704 (15)	0.0213 (12)	0.0374 (13)	0.0138 (11)
S2	0.0561 (4)	0.0505 (4)	0.0443 (4)	0.0073 (3)	0.0089 (3)	0.0258 (3)

### Geometric parameters (Å, º)

**Table:**

C1—C6	1.379 (4)	C15—C20	1.381 (3)
C1—C2	1.385 (4)	C15—C16	1.394 (3)
C1—S1	1.766 (3)	C15—S2	1.771 (3)
C2—C3	1.380 (4)	C16—C17	1.370 (4)
C2—N2	1.462 (4)	C16—N4	1.474 (3)
C3—C4	1.380 (5)	C17—C18	1.381 (4)
C3—H3	0.9300	C17—H17	0.9300
C4—C5	1.366 (5)	C18—C19	1.372 (4)
C4—H4	0.9300	C18—H18	0.9300
C5—C6	1.379 (4)	C19—C20	1.383 (4)
C5—H5	0.9300	C19—H19	0.9300
C6—H6	0.9300	C20—H20	0.9300
C7—O3	1.214 (3)	C21—O8	1.210 (3)
C7—N1	1.395 (3)	C21—N3	1.395 (3)
C7—C8	1.487 (4)	C21—C22	1.486 (4)
C8—C13	1.380 (4)	C22—C27	1.384 (4)
C8—C9	1.384 (4)	C22—C23	1.389 (4)
C9—C10	1.381 (4)	C23—C24	1.379 (4)
C9—H9	0.9300	C23—H23	0.9300
C10—C11	1.378 (4)	C24—C25	1.384 (4)
C10—H10	0.9300	C24—H24	0.9300
C11—C12	1.374 (4)	C25—C26	1.376 (4)
C11—C14	1.505 (4)	C25—C28	1.503 (4)
C12—C13	1.386 (4)	C26—C27	1.382 (4)
C12—H12	0.9300	C26—H26	0.9300
C13—H13	0.9300	C27—H27	0.9300
C14—H14A	0.9600	C28—H28A	0.9600
C14—H14B	0.9600	C28—H28B	0.9600
C14—H14C	0.9600	C28—H28C	0.9600
N1—S1	1.641 (2)	N3—S2	1.642 (2)
N1—H1N	0.855 (10)	N3—H3N	0.853 (10)
N2—O4	1.196 (4)	N4—O10	1.216 (3)
N2—O5	1.207 (4)	N4—O9	1.216 (3)
O1—S1	1.419 (2)	O6—S2	1.430 (2)
O2—S1	1.429 (2)	O7—S2	1.420 (2)
			
C6—C1—C2	118.7 (2)	C20—C15—C16	118.1 (2)
C6—C1—S1	117.4 (2)	C20—C15—S2	118.89 (19)
C2—C1—S1	123.8 (2)	C16—C15—S2	122.58 (19)
C3—C2—C1	120.9 (3)	C17—C16—C15	122.1 (2)
C3—C2—N2	116.8 (3)	C17—C16—N4	116.7 (2)
C1—C2—N2	122.3 (3)	C15—C16—N4	121.2 (2)
C2—C3—C4	119.1 (3)	C16—C17—C18	118.6 (2)
C2—C3—H3	120.4	C16—C17—H17	120.7
C4—C3—H3	120.4	C18—C17—H17	120.7
C5—C4—C3	120.6 (3)	C19—C18—C17	120.8 (3)
C5—C4—H4	119.7	C19—C18—H18	119.6
C3—C4—H4	119.7	C17—C18—H18	119.6
C4—C5—C6	119.9 (3)	C18—C19—C20	120.1 (3)
C4—C5—H5	120.0	C18—C19—H19	120.0
C6—C5—H5	120.0	C20—C19—H19	120.0
C1—C6—C5	120.6 (3)	C15—C20—C19	120.4 (2)
C1—C6—H6	119.7	C15—C20—H20	119.8
C5—C6—H6	119.7	C19—C20—H20	119.8
O3—C7—N1	120.4 (2)	O8—C21—N3	119.8 (3)
O3—C7—C8	123.6 (3)	O8—C21—C22	123.9 (2)
N1—C7—C8	116.0 (2)	N3—C21—C22	116.3 (2)
C13—C8—C9	118.8 (3)	C27—C22—C23	118.3 (3)
C13—C8—C7	123.2 (2)	C27—C22—C21	123.5 (2)
C9—C8—C7	118.0 (2)	C23—C22—C21	118.1 (3)
C10—C9—C8	119.9 (3)	C24—C23—C22	120.2 (3)
C10—C9—H9	120.0	C24—C23—H23	119.9
C8—C9—H9	120.0	C22—C23—H23	119.9
C11—C10—C9	121.8 (3)	C23—C24—C25	122.0 (3)
C11—C10—H10	119.1	C23—C24—H24	119.0
C9—C10—H10	119.1	C25—C24—H24	119.0
C12—C11—C10	117.8 (3)	C26—C25—C24	117.3 (3)
C12—C11—C14	121.0 (3)	C26—C25—C28	120.9 (3)
C10—C11—C14	121.2 (3)	C24—C25—C28	121.8 (3)
C11—C12—C13	121.3 (3)	C25—C26—C27	121.8 (3)
C11—C12—H12	119.3	C25—C26—H26	119.1
C13—C12—H12	119.3	C27—C26—H26	119.1
C8—C13—C12	120.4 (3)	C26—C27—C22	120.5 (3)
C8—C13—H13	119.8	C26—C27—H27	119.7
C12—C13—H13	119.8	C22—C27—H27	119.7
C11—C14—H14A	109.5	C25—C28—H28A	109.5
C11—C14—H14B	109.5	C25—C28—H28B	109.5
H14A—C14—H14B	109.5	H28A—C28—H28B	109.5
C11—C14—H14C	109.5	C25—C28—H28C	109.5
H14A—C14—H14C	109.5	H28A—C28—H28C	109.5
H14B—C14—H14C	109.5	H28B—C28—H28C	109.5
C7—N1—S1	123.28 (19)	C21—N3—S2	123.47 (19)
C7—N1—H1N	121 (2)	C21—N3—H3N	122 (2)
S1—N1—H1N	115 (2)	S2—N3—H3N	113.1 (19)
O4—N2—O5	124.6 (3)	O10—N4—O9	124.0 (2)
O4—N2—C2	118.1 (3)	O10—N4—C16	118.3 (2)
O5—N2—C2	117.2 (3)	O9—N4—C16	117.5 (2)
O1—S1—O2	119.62 (13)	O7—S2—O6	119.71 (14)
O1—S1—N1	109.89 (13)	O7—S2—N3	110.46 (13)
O2—S1—N1	104.51 (12)	O6—S2—N3	104.21 (12)
O1—S1—C1	106.60 (13)	O7—S2—C15	107.13 (13)
O2—S1—C1	109.45 (13)	O6—S2—C15	107.80 (12)
N1—S1—C1	106.06 (12)	N3—S2—C15	106.88 (11)
			
C6—C1—C2—C3	0.4 (4)	C20—C15—C16—C17	1.0 (4)
S1—C1—C2—C3	177.3 (2)	S2—C15—C16—C17	−171.2 (2)
C6—C1—C2—N2	179.0 (3)	C20—C15—C16—N4	−176.5 (2)
S1—C1—C2—N2	−4.1 (4)	S2—C15—C16—N4	11.3 (3)
C1—C2—C3—C4	1.5 (4)	C15—C16—C17—C18	−1.3 (4)
N2—C2—C3—C4	−177.2 (3)	N4—C16—C17—C18	176.3 (2)
C2—C3—C4—C5	−2.1 (5)	C16—C17—C18—C19	0.1 (4)
C3—C4—C5—C6	0.9 (5)	C17—C18—C19—C20	1.3 (4)
C2—C1—C6—C5	−1.6 (4)	C16—C15—C20—C19	0.5 (4)
S1—C1—C6—C5	−178.7 (2)	S2—C15—C20—C19	173.0 (2)
C4—C5—C6—C1	0.9 (5)	C18—C19—C20—C15	−1.7 (4)
O3—C7—C8—C13	164.3 (3)	O8—C21—C22—C27	157.4 (3)
N1—C7—C8—C13	−17.4 (4)	N3—C21—C22—C27	−21.4 (4)
O3—C7—C8—C9	−15.1 (4)	O8—C21—C22—C23	−18.9 (4)
N1—C7—C8—C9	163.2 (3)	N3—C21—C22—C23	162.3 (2)
C13—C8—C9—C10	0.1 (4)	C27—C22—C23—C24	0.7 (4)
C7—C8—C9—C10	179.5 (3)	C21—C22—C23—C24	177.1 (3)
C8—C9—C10—C11	−0.4 (5)	C22—C23—C24—C25	−1.0 (5)
C9—C10—C11—C12	0.7 (5)	C23—C24—C25—C26	0.8 (5)
C9—C10—C11—C14	−178.8 (3)	C23—C24—C25—C28	−177.8 (3)
C10—C11—C12—C13	−0.8 (4)	C24—C25—C26—C27	−0.3 (5)
C14—C11—C12—C13	178.8 (3)	C28—C25—C26—C27	178.3 (3)
C9—C8—C13—C12	−0.1 (4)	C25—C26—C27—C22	0.0 (5)
C7—C8—C13—C12	−179.5 (2)	C23—C22—C27—C26	−0.2 (4)
C11—C12—C13—C8	0.5 (4)	C21—C22—C27—C26	−176.4 (3)
O3—C7—N1—S1	−3.1 (4)	O8—C21—N3—S2	−12.4 (4)
C8—C7—N1—S1	178.58 (18)	C22—C21—N3—S2	166.47 (19)
C3—C2—N2—O4	114.8 (3)	C17—C16—N4—O10	55.5 (3)
C1—C2—N2—O4	−63.9 (4)	C15—C16—N4—O10	−126.9 (3)
C3—C2—N2—O5	−62.0 (4)	C17—C16—N4—O9	−120.9 (3)
C1—C2—N2—O5	119.3 (3)	C15—C16—N4—O9	56.8 (3)
C7—N1—S1—O1	−50.4 (3)	C21—N3—S2—O7	60.8 (3)
C7—N1—S1—O2	−180.0 (2)	C21—N3—S2—O6	−169.4 (2)
C7—N1—S1—C1	64.4 (2)	C21—N3—S2—C15	−55.4 (2)
C6—C1—S1—O1	19.2 (2)	C20—C15—S2—O7	−8.7 (2)
C2—C1—S1—O1	−157.8 (2)	C16—C15—S2—O7	163.4 (2)
C6—C1—S1—O2	149.9 (2)	C20—C15—S2—O6	−138.8 (2)
C2—C1—S1—O2	−27.1 (3)	C16—C15—S2—O6	33.3 (2)
C6—C1—S1—N1	−97.9 (2)	C20—C15—S2—N3	109.7 (2)
C2—C1—S1—N1	85.1 (2)	C16—C15—S2—N3	−78.2 (2)

### Hydrogen-bond geometry (Å, º)

**Table:**

*D*—H···*A*	*D*—H	H···*A*	*D*···*A*	*D*—H···*A*
N3—H3*N*···O2	0.85 (1)	2.30 (1)	3.141 (3)	168 (3)
